# Demographic characteristics of convicted child sexual abusers in South of Sweden, between 2013 and 2018: a pilot study

**DOI:** 10.1080/20961790.2022.2052590

**Published:** 2022-11-04

**Authors:** Emelie Stiernströmer, Marie Väfors Fritz, Caroline Mellgren, Ardavan Khoshnood

**Affiliations:** a Department of Criminology, Malmö University, Malmö, Sweden; b Department of Clinical Sciences Lund, Skane University Hospital, Emergency Medicine, Lund University, Lund, Sweden

**Keywords:** Child sexual abuse, child rape, child molestation, demographic characteristics, frequency analysis, offender characteristics

## Abstract

This study evaluates variables concerning demographic characteristics for all adult male offenders convicted of Child Sexual Abuse (CSA) aged 0–17 in Malmö, Sweden between 2013 and 2018. All convictions (*n* = 18) based on court documents from the District Court, the Court of Appeals and information from the Swedish Tax Agency were reviewed. A total of 30 victims were identified. Frequency analyses show that the most common features were that of a single offender, averaging 25 years old, with a non-Swedish background and a high school degree. The predominately extrafamilial CSA (i.e. committed by an acquaintance to the family) occurred in a private setting and consisted of penetrative acts. Girls averaging 13 years old were abused multiple times, under fear and pressure. Although assumptions based on these results are preliminary, they provide a clearer image of the typical circumstances under which CSA occurred within this time frame and geographical location. This study is a first attempt to construct an overview of demographic characteristics of CSA. As more data are gathered from this region, more sophisticated analyses can be conducted, providing stronger generalizations. Information of this kind may be important for research, classification of offender profiling and in case linking.

## Background

Child Sexual Abuse (CSA) encompasses many types of sexually abusive acts towards children. It is a general term describing a wider range of events that vary in demographic characteristics such as the victim’s age of onset, relationship between the child and the perpetrator, abuse quantity, degree of contact and use of force. There are many definitions of CSA in use [1], each of which may have subtle differences in cover or terminology that influence surveillance and reporting effects and potentially leading to different policy, service or legal implications. Nonetheless, this type of crime is considered a global problem [2, 3]. For the child victims, the consequences of CSA may have both short-term and grave life-long consequences, e.g. increased risk of experiencing psychosexual distress, health-risk behaviours, interpersonal problems and psychopathology [4–12]. Children may also be particularly vulnerable to the negative effects stemming from the exposure to sexual abuse. One of the reasons for this is that the offences occur at a time when the victims are in the process of developing their identity, their capacities for trust and intimacy, and also their sexuality. The consequences of sexual offences may thus be even more extensive and serious for children than they are for adults [13].

In Sweden, and according to the Swedish National Council for Crime Prevention (BRÅ), reports on CSA continue to increase [14, 15]. Although improving and updating our knowledge about CSA may not be enough to hinder it from occurring, it may provide a better understanding of the main features of this particular crime. One way to gain this type of insight is to focus on recently committed CSA cases, by reviewing the demographic characteristics of the offenders, the crime and the victims. Who commits these crimes, under what circumstances and towards whom are questions that may constitute imperative knowledge that could be used for research purposes [16, 17]. When a growing amount of data has been compiled, it can also be used in criminal investigations by the police. However, despite the popular concern and increased public awareness of CSA, portraying the demographic characteristics of this crime in modern-day Sweden has attracted limited scientific interest and thus little research on CSA in Sweden has been conducted.

### Definition of child sexual abuse and child rape in Swedish law

In 2005, the Swedish sex crime legislation was altered in a number of important ways. One of the important changes concerned the nature of the threat and violence required for a sexual offence to be defined as rape. The most significant change involved the introduction of a new offence in the penal code, Section 4, Chapter 6, specified as *rape of a child*. This new rape provision relates to all cases where the victim is under the age of 15 (i.e. the Swedish age of sexual consent), irrespective of the nature of the relationship between victim and offender. The new rape provision relates to all the cases involving victims aged 15 to 17 if the perpetrator is the victim’s parent, guardian or similar. The sexual acts covered by this new offence are the same as those covered by the rape statute as specified in the 1998 version, i.e. vaginal, anal or oral intercourse or penetrative acts involving objects or similar. One important difference compared to the earlier formulation of the rape statute is that there is no requirement for any form of threat or violence to have been exerted in relation to the child in order for the act to be defined as rape. In principle, the new statute defines rape as any form of sexual intercourse, or any of the other sexual acts viewed as being equivalent to sexual intercourse, involving a child under the age of 15, irrespective of the actions of the offender in other respects. Concerning children aged 15 to17 in cases where the offender is not a parent, guardian or similar, the legislative changes of 2005, implied a reduction in the seriousness of the threats and violence required to be criminally liable for a rape offence. In a similar manner, the type of threats needed for an offender to become criminally liable for the rape offence was also changed: from the requirement that threats must represent an immediate danger to the child’s life or health, to a requirement that the offender has threatened the child to commit any form of criminal acts. Although CSA can be distinguished from child rape (e.g. in terms of severity), the juridical penalty act for the two remains the same. Hence, rape of a child is included under the banner of CSA. For the purpose of the current study, CSA will hereforth be used to refer to both crime types (child sexual abuse and rape of a child).

### The Swedish statistics

It is widely accepted that many CSAs go unreported, hence there is a large “dark number” for this crime [18, 19]. However, in parallel to the increasing attention in society towards children’s exposure to sexual abuse, the management of the Swedish criminal law of sexual crimes towards children has increased since the 1980s. More crimes of this type are reported to the police, which requires many new demands by the juridical system and the police [5].

Irrespective of victim age, the total number of rapes reported to the police in Sweden (*n* = 7 370) increased with 10% (*n* = 730) from 2016 to 2017. Of the total number (*n* = 7 370) of reported rapes in 2017, 13% (*n* = 958) constituted rape against a child 15 to 17 years old and 29% (*n* = 2 137) rape against a child under the age of 15.

During 2018 there were 22 500 sex crimes reported in Sweden. This constitutes an increase by 2% (*n* = 485) as compared to 2017. Concerning reported rapes, these increased by 8% (*n* = 7 960). Of these reported rapes, 44% (*n* = 3 490) constituted rape against a child aged 0 to 17. This is an increase with 12% (*n* = 395) as compared to 2017, and an increase with 20% (*n* = 591) since 2009. Of the reported rapes against a child 0 to 17 during 2018, 91% (*n* = 3 176) was against a girl and 9% (*n* = 314) against a boy.

Based on the 2008 Swedish crime statistics, a report from BRÅ [5] shows that the majority of the CSA reported to the police against the youngest girls (0 to 11 years old) were committed within the family (i.e. interfamilial abuse) or by an acquaintance to the family (i.e. extrafamilial abuse). For victims aged 0 to 11 years old (*n* = 88), the suspect was a person from either the immediate or extended family in 71% of the cases (*n* = 62), followed by friends or acquaintances in 26% of the cases (*n* = 23). Non-acquaintances only constituted 3% (*n* = 3). The offenders suspected of CSA were also considerably older than their victims.

During 2020, there were 9 580 rapes reported in Sweden, of which 45% (*n* = 4 300) were labeled rape against a child aged 0–17 years old. This constitutes an increase by 21% (*n* = 756) crimes since 2019 and an increase by 44% (*n* = 1 310) since 2011. Of the total reported rapes against a child during 2020, 90% (*n* = 8 622) were crimes against a girl and 10% (*n* = 958) against a boy [14].

Although the above information reported in the different reports by BRÅ is informative, the data they provided mainly concern crimes reported to the police and not convictions *per se*, and mainly concerns rape allegations. The above reports by BRÅ are less informative regarding central demographic characteristics of the offenders convicted of CSA (including but not limited to rape), the crime and the victims, and concern all of Sweden. At the current moment it is therefore not possible to distinguish and/or relate characteristics of successful CSA conviction between certain cities. Only in one report [20] have sex crimes been compared between Sweden’s three largest cities, however, the reports presented above did not differentiate between sex crimes against adults and CSA. It is hence unknown whether sex crimes in general and CSA in particular differ between these cities that have different geographical positions which may entail different crime issues. For instance, Malmö and the southern region of Sweden is most affected by firearm-related violence in Sweden and this part also has had serious problems with homicide [21, 22]. The current understanding of what constitutes the typical circumstances of CSA in different parts of Sweden as well as in Sweden as a whole, can therefore be said to remain unclear. Gathering more information on the perpetrators of CSA can be important both in terms of research (e.g. studying whether crime patterns change over time) and for investigation purposes. With this said, there are also concerns when gathering data on perpetrators, such as misunderstandings and misuse of the information. Any such attempt must therefore be done with caution.

### Type of sex offenders

The most frequently used and empirically tested classification (including demographic characteristics) of sex crimes is based on victim age and distinguish between rapists (i.e. those who abuse adults) and offenders of CSA [23, 24]. CSA is a crime, in which the dynamics of the crimes (e.g. when, where and how) may differ from adult sexual abuse [25, 26]. For instance, rapists are often characterized as more antisocial, psychopathic and hostile than child molesters [25]. Therefore, CSA should not be handled in the same way as adult sex abuse. In addition, in CSA the offender is often older than the rapists [25, 27]. Studies also suggest that rapists are found to be less educated and more likely to have been in a relationship than offenders of CSA [25]. If different subgroups of sex offenders (for example rapists versus offenders of CSA) present different neuropsychological profiles, as discussed by Joyal et al. [28], this could at least in part help explain these differences.

Like all sexual offenders, CSA offenders constitute a heterogenous group. They are hence difficult to classify as they, for instance, vary in economic status, gender, marital status, ethnicity (and/or nationality) and sexual orientation [27]. They have also been categorized based on their relationship to the victim. Intrafamilial CSA offenders, e.g. incest offenders/abuse within the family, appear to be less likely victimizing males, cause less physical injury, are less likely to exhibit pedophilia and have lower sexual and violent recidivism rates [29].

Victims of intrafamilial CSA are also typically younger than extrafamilial abuse victims [30] and this type of CSA can be disproportionally difficult to prevent, identify and prosecute [10]. Although in intrafamilial CSA offenders substitute a child for an adult sexual partner, the offenders often maintain their adult sexual relationships [31, 32]. Importantly, although certain demographic characteristics have been found to be associated with intra- and/or extrafamilial CSA, other associations may exist and hence should not be ignored or dismissed.

### The current study

The current understanding of CSA in Sweden is inadequate. One way to increase the understanding regarding what type of CSA that is typically occurring in today’s society is to portray the main features (using frequency and percentage) of convicted offender, their crime and their victims by reviewing court documents and police reports—a common and validated method [16, 33, 34]. The sample herein examined is restricted to all CSA cases by an adult male leading to a conviction in Sweden’s third largest city, Malmö, between 2013 and 2018. The most commonly used features (in studies on sex crimes) are examined in an attempt to portray some of the demographic characteristics (in frequency and percentage) of the typical circumstances under which recent CSAs have occurred.

## Materials and methods

### Material

All cases of adult male offenders convicted of CSA (children aged 0 to 17) in Malmö were included in the study. The restriction to male offenders was partly because few convicted female rapists were to be expected as females are more often victimized [14]. Another reason was the limited existing knowledge of female offenders that targets children and the significant differences between male and female sexual abusers [27, 35].

The study was approved by the local regional ethics review board in Lund (Dnr 2018/801). An opt-out model of informed consent was employed, where data were collected and used automatically unless the offender actively dissented, following information posted on the university website concerning the study. No one withdrew from the study.

Based on the Swedish Penal Code, Chapter 6 (unofficial translation) the crime classifications for the CSA cases in the current study included the following appellations: sexual exploitation of children (incl. attempted or preparation of (and/or) aggravated sexual exploitation of children), child molestation, child pornography (incl. attempted or preparation of (and/or) aggravated child pornography), purchase of a sexual act of a child, attempt or preparation of sexual assault of a child, and child rape (incl. attempted or preparation of aggravated child rape, (incl. aggravated child rape)). All available court file documents from the District Court of Malmö were reviewed, along with cases that were appealed to the Scania and Blekinge Court of Appeal. Due to inconsistencies in how the offenders’ nationality was stated in the court documents, the information was obtained from the Swedish Tax Agency. A total of 18 cases were collected from the District Court in Malmö (14 cases included multiple sex charges against a child). In 11 cases, the conviction included rape of a child, attempted rape of a child or aggravated rape of a child.

### Variables

The demographic characteristics of interest concerned the offenders, the crime, and the victims. The variables chosen for this study were selected due to their being commonly used in this and similar lines of research [16, 24, 36–38]. All variables were coded in a numerical manner according to the number of categories for each variable; as an example, age was broken down into four categories and coded as “1” for those between 18 and 20 years old, “2” for those between 20 and 30 years old.

The offender demographics comprised six variables: age group (15–18, 19–20, 21–30, 31–40, 41–50), nationality (non-Swedish, Swedish), civil status (single/divorced, relationship/married), educational level (elementary school, high school, folk high school/university, unknown), socioeconomic status (SES: in school, employed, unemployed), and victim frequency, meaning how many victims the offender abused at one point (one, two, three or more). To calculate the offender age group, the date of birth and conviction/sentence date was used. An offender’s nationality was, in accordance with BRÅ’s definition [39], classified as “non-Swedish” if the offender had at least one parent from a foreign country or was born abroad. In other cases, they were defined as Swedish.

The crime characteristics comprised six variables: offender-victim age difference (0–5, 6–10, 11–20, 21–30, 31–40), offender-victim relationship (none, intrafamilial, extrafamilial), penetration act (yes, none), manipulation approach (secret/play, explicit threats, fear/pressure), physical violence (yes, no), and crime location (private, public). Concerning the victim-offender relationship, intrafamilial relationships included all biological relatives and stepfamily. Extrafamilial relationships were acquaintances outside the family. Penetration act referred to whether the offender in some way penetrated the victim (e.g. vaginal, anal and oral intercourse or penetration using fingers/objects). A “yes” indicated penetration by the offender. “No” indicated only sexually touching, i.e. non-penetrative. Coercive control concerned in what way the offender controlled the child (e.g. told them the abuse was a game, or a secret). For physical violence, “yes” implied that the offender hit, kicked or inflicted serious bodily harm to the victim. Crime location contained two categories: public or private. Examples of public crime scenes were e.g. public toilet or a pre-school, while abuse that took place on private property (e.g. inside house or car) was defined as within the private sphere.

The victim demographics comprised three variables: gender (female, male), conviction age group (0–5, 6–10, 11–15, 16–18), and abuse occurrences per victim (single, multiple abuse). As with offender age, the date of birth and conviction date were used to calculate the age of the victim.

### Procedure

Following contact with the District Court, a list of the convicted offenders was created and their court documents compiled dating back 5 years. This time period was chosen based on the cycle of operation at the country’s District Courts, meaning that all the material held by the courts can be eliminated after 5 years, incl. pre-trial material. In addition, the District Court’s computer system registry can only search 5 years back in time. Thereafter cases are no longer searchable by the registry. Each case was reviewed and checked against the Court of Appeals to check if the decision by the District Court was appealed by the defendant or the prosecutor. After coordination with the District Court, the Court of Appeals, and the Swedish Tax Agency, 18 cases were identified where at least one offender was convicted of CSA.

### Data treatment

Once the reports and documents were gathered, a systematic approach using document analysis was conducted [40]. This included principles of decontextualization in which relevant parts of the documents were removed from its context and studied in combination with text related to the same phenomena [41]. The step allowed us to study patterns (incl. individual patterns) as a phenomenon, using a matrix of similar characteristics [40, 42]. Thereafter the text content was encoded into numbers and exported to SPSS V.24 (IBM Corp., Armonk, NY, USA). Due to the limited number of offenders, frequency analyses were conducted to determine the distribution of the selected demographic characteristics concerning the offenders, the crime and the victims.

## Results

Table 1 presents the frequency distribution and percentages for the demographic characteristics. At conviction date, the average age for the offenders is 24.6 years old (SD = 10.32, range 16–49). The average age for the victims is 13.1 years old (SD = 5.11, range 4–26), The average age difference at the time of sentencing between the offenders and the victims is 13.4 years (SD = 11.1, range 2–36).

**Table 1.** Frequency distribution (and percentage) of demographic characteristics of Child Sexual Abuse (CSA) in Malmö, Sweden, 2013 to 2018.

Offender demographics (*n* = 18)			Crime characteristics (*n* = 30)			Victim demographics (*n* = 30)		
Variable	Number	Frequency (%)^a^	Variable	Number	Frequency (%)^a^	Variable	Number	Frequency (%)
Offender age group (years)^b^			Offender-victim age difference (years)^b^			Gender		
18 or below	5	28	0–5	8	27	Male	7	23
19–20	3	17	6–10	12	40	Female	23	77
21–30	6	33	11–20	2	7	Victim age group (years)^b^		
31–40	2	11	21–30	5	17	5 or below	3	10
41–50	2	11	31–40	3	10	6–10	4	13
Nationality			Offender-victim relationship			11–15	17	57
Swedish	6	33	None	7	23	16–18	4	13
Non-Swedish	12	67	Intrafamilial	6	20	18 or above	2	7
Civil status			Extrafamilial	17	57	Abuse occurrence per victim		
Single/divorced	12	67	Penetration act			Single	13	43
Relationship/married	6	33	Yes	18	60	Multiple	17	57
Education level			No	12	40			
Elementary school	3	17	Coercive control					
High school	12	67	Secret/play	6	20			
Higher education	1	6	Explicit threats	9	30			
No record	2	11	Fear/pressure	15	50			
Socioeconomic status			Physical violence					
In school	9	50	Yes	6	20			
Employed	6	33	No	24	80			
Unemployed	3	17	Crime location					
Victim frequency			Private	20	67			
One	9	50	Public	10	33			
Two	7	39						
Three or more	2	11						

a Numbers are rounded so the percentages may not add up to 100%.

b The age group variable for CSA offenders and the victims are both based on birth date and date of conviction.

Regarding the nationality of the offenders, 12 were of non-Swedish origin whereas six of the 18 offenders had a Swedish nationality. The majority (*n* = 12) were either single or divorced. Only six were currently in a relationship or married when the offence took place. Most of the offenders (*n* = 12) were either currently in high school or had a high school education as their highest education, three offenders had an elementary school education whereas only one had a higher education. The education level of two offenders remained “unknown”. Concerning the socioeconomic status, nine offenders were currently in school, and were therefore not employed, six offenders held a job at the time of the crime and three offenders were unemployed. Regarding victim frequency, most of the offenders (*n* = 9) were convicted for abusing only one child. Second most common (*n* = 7) was that the offenders had abused two children, whereas abusing three or more children was the least common (*n* = 2).

Concerning offender-victim relationship, 17 cases were extrafamilial abuse. The remaining cases either had no relationship between the offender and the victim (*n* = 7) or were labeled intrafamilial abuse (*n* = 6). Of all the cases (*n* = 30), the majority included penetration (*n* = 18) whereas 12 cases did not include penetration. Regarding coercive control, half of all the cases reported that fear and/or pressuring of the child had been used (*n* = 15), nine cases included explicit threats and six cases included secrets or other secret games to lure the child. The majority of cases reported no physical violence (*n* = 24) whereas six cases explicitly stated that this was used during the crime. Most of the crimes were located in private (*n* = 20), whereas 10 were in public places.

Concerning the victims, the majority of children were female (*n* = 23), whereas seven were boys. The majority of cases reported that they were abused multiple times by their abuser (*n* = 17), whereas 13 cases reported only being abused once.

## Discussion

This study aimed to portray the distributions of selected variables concerning all convicted CSA cases that occurred in Malmö between 2013 and 2018. Although this study is based on frequency data of only 18 offenders, a better picture of this crime type still emerged.

In line with previous studies, our findings suggest that the crime dynamics of CSA may be dissimilar than that of adult rape, at least within the framework herein provided. However, in contrast to the common understanding that CSA offenders are older than rapists [25, 27], the findings presented here may be indicative of the opposite pattern. When compared to convicted rapists in discussing adult females in Sweden using the same geographical location, time window and definition of age, CSA offenders are younger, averaging 24.6 years old, whereas rapists averaged 33 years old [37]. The CSA offenders in the current study are also younger than the offenders of CSA examined in other countries [24] reporting an average offender age of 37.7 and 33.7 for rapists (see also [43–45]). However, in contrast to the current study, the victims examined by Simons et al. [24] were children aged 13 years old or younger.

Another finding concerned the nationality of the offenders in which 67% had a non-Swedish background. Comparing offender ethnicity or nationality across studies is very difficult as the definitions may vary greatly. Any explanation for why young non-Swedish men primarily committed these sex crimes are therefore speculative at best. The higher concentration of offenders with foreign backgrounds is however supported by Stiernströmer et al. [37], examining the characteristics of convicted adult rapists, using the same definition, within the same period and geographical location. It is also likely that sociodemographic features, such as nationality, reflect generally biased arrest rates. This may in part be explained by recent statistics from Statistics Sweden which states that Malmö has a high concentration of individuals of foreign background [46]. For instance, one third of the nationally registered citizens in Malmö are foreign-born citizens (as compared to one fourth of the citizens in Stockholm and Gothenburg).

In regards to the offender’s civil status, the findings presented here suggest that the predominately extrafamilial CSA were committed by primarily single males. In the current case their civil status may be explained by their young age. The offender’s civil status, herein examined, is furthermore in line with results on adult rape characteristics using a Swedish sample [37] as well as with international studies [24, 45].

The age difference between the offender and his victim indicates that the majority of the relationships had an age span of 0 to 10 years. Given the mean victim age (13 years old), these numbers are fairly in line with previously known information from BRÅ [5] yet here showing a slightly wider age span. This difference may be due to BRÅ only examining rape crimes reported to the police (rather than convictions of CSA *per se*, which includes but is not limited to child rape) within all of Sweden (rather than a particular city). In this context it is also important to consider the number of incidents of sexual abuse that accrued but was not reported.

The victims herein examined were predominately girls, whereas boys constituted 23% (*n* = 7), and the abuse took place indoors behind closed doors and contained some act of penetration [5, 14, 24]. Our data show that repeated sexual abuse was relatively common, and that offender-victim relationship was mostly that of extrafamilial nature. This is in contrast to the pattern reported by BRÅ that most CSA are committed by family members [5]. Part of the explanation for this difference may concern definitions. It may also stem from greater support from the victim for prosecution which may have an impact on the conviction rate [47].

Approximately 20% of the victims studied were physically injured by the offender during the course of the crime. What explains the larger proportions of non-physical violence in this study may be due to the variables that this study did not measure. Further studies must examine whether intrafamilial CSA and fewer physical injuries are correlated [29] as well as whether the presence of threats or physical violence are factors that increased with the age of the victim (e.g. [5]), something that this study is unable to confirm. However, the younger children in this sample may have been more easily controlled and hence less force was needed to control them. This may explain the larger proportion of non-physical violence reported in this study.

## Limitations

All studies are associated with methodological issues of different kinds. This study is no exception. With the current dataset the most thinkable starting point concerning the purpose, as in this case, is to increase the knowledge on CSA. There are therefore important limitations that should be considered when interpreting these findings. This study focused exclusively on CSA convictions that were not randomly selected. However, focusing on convictions is a reasonable decision when studying the offender, crime, and victim characteristics, as much of this information may be unknown in cases that were reported to the police but did not lead to a conviction. Yet a risk remains that cases leading to a conviction differ in the offender and crime characteristics from cases that did not. For instance, there may be an overrepresentation of inexperienced rather young offenders who are not forensically aware, as compared with those that were not convicted and therefore not identified in the present study.

There are also limitations regarding the variables. Offender and victim age were measured at conviction date. Hence, the exact age of the offender and the victim at the time of the crime remains uncertain. Conviction date was used since the documents and police reports often did not specify the exact time of the first crime. In CSA, the abuse may have been occurring for a long time before the child builds up the courage to disclose the abuse to an adult. Consequently, conviction age formed the best approach to characterize age.

Concerning the variables, it is also important to keep in mind that any assumptions based on these frequency data are limited to the variables included in this study. Unfortunately, the study was limited in which variables to include, based on the information given in the documents from the courts and the police.

Some variables were also broadly defined. For instance, an offender would be classified as a foreigner if he was born outside of Sweden. This definition of nationality explains little about the actual origin of the majority of these offenders. One problem with using the term nationality is that the offenders may be of Swedish nationality but still have a foreign background. This was however the definition granted for this study by the ethics committee, and previously used by other government agencies (e.g. [39]). We therefore stress the importance of being reflective of such demographic data when alluding that foreigners may be a characteristic of CSA. The outcome of the current study is not stating that non-natives are statistically more likely to commit these crimes. Hence, being a foreigner is not a risk factor for CSA and/or child rape. This study merely informs that among the demographic characteristics examined in the frequency analysis, non-native offenders charged for committing CSA were overrepresented.

Other important limitations concern the missing data and the small sample size which affect the assumptions based on these results. We emphasize that it is with caution one should draw any reliable conclusions on the basis on the given data. In addition, characteristics of the convicted offenders are not just resulting from the offenders themselves, but also from the large dark number of sexual abuses that are never reported to the police or processed in court and therefore not reflected by the present study. This may contribute to explain why foreigners are more often reported than the Swedish people. There were also issues with victims reporting their offenders, especially when the victims are likely to be related to the offender, which adds to the complexity.

The small sample size may be particularly challenging given the heterogeneity that exists between sex offenders. The small sample size also prevented us from running more complex statistical analysis on the dataset. One must therefore be very considerate when generalizing these findings which are only based on frequency data. However, a small sample size is, for practical reasons, common in criminal research, especially when focusing on crimes that occurred in a limited geographical location and within a limited time frame, as in the current study.

## Conclusions and future direction

Notwithstanding these limitations, this study makes a novel contribution to the Swedish CSA literature by being the first examining and categorizing some of the demographic characteristics of recently convicted offenders in the South of Sweden. The fact that there is currently no recent Swedish study on CSA that can be used as a comparison stresses the need for more studies to be conducted on this matter.

It should also be emphasized that this study should be considered preliminary at best. As more data will be available every 5 years, it may be possible to construct a database in which new information on sexual abuse characteristics will be added, including both adult sex offences and CSA. Over the years, as a larger sample size becomes available, more sophisticated and informative analyses can be conducted, and stronger generalizations can be made. This may allow tracking changes in CSA patterns over time, reflecting which characteristics remain stable and which fluctuates. It also allows comparisons to be made between convicted adult sex offenders and CSA offenders. This information could be used not only for research purposes but may also with time help identify different clusters of offenders that can be used in offender profiling [16] and in case linking [17].

The overall aim of this research and others like it would be to transfer knowledge and skills based on research over to the groups in society that can use them for preventative purposes and for clinicians whose task is to effectively assess and treat sexual offending. However, constructing a future database of this kind is not without problems as it may be misinterpreted. For instance, age and nationality may not be legitimate variables to judge whether one is a CSA or not. In addition, rape and other sexual abuse crimes against children have been suggested to occur for different reasons, and as a result of a complex interplay of many different factors, risk factors within the offender, and the victim and in the environment [48]. It is therefore emphasized that more studies are needed before any conclusions can be drawn.

Future studies should attempt to replicate and extend on this study to see whether the findings are comparable to CSA in other Swedish as well as Scandinavian cities. They should attempt to define more precisely the study population and include a comparison or control group. The over-representation of offenders with non-Swedish background in our sample should also be investigated in future studies which may use other definitions that describe their origin in a more detailed manner. They should attempt to understand the possible significance of potentially differing sociocultural factors, e.g. views and values related to gender and sexuality. It may also be useful to differentiating CSA according to age of the victim and motivation, i.e. pedophilia *vs*. compensatory rape (e.g. [32]).

Overall, in an attempt to portray the patterns of CSA in Malmö, the following can be inferred from our findings: the typical CSA offender is averaging 25 years old, of non-Swedish descent, single, with a lower educational level and currently in school. He victimizes and penetrates girls, averaging 13 years old, repeats the abuse often behind closed doors. Their association is that of extrafamilial nature exclusive of physical violence. Despite the problems that are associated with this study, such as the fact that the data are based on frequency data of 18 offenders, the study still allows for a relatively good overview of the picture of this crimes type. It provides a clearer and more updated picture of the typical circumstances of the typical CSA cases with a successful conviction that occurred in the South of Sweden within the past 5 years.
